# Case of Remarkable Organic Disease of the Stomach and Neighbouring Parts, with the Appearances on Dissection

**Published:** 1816-08

**Authors:** Alexander Dewar

**Affiliations:** Surgeon of H. M. S. Boyne, Flag Ship of Admiral Lord Exmouth


### Medico-Chirurgical

### JOURNAL AND REVIEW.

VOL. II.] AUGUST, 1816. [NO. 8.

### PART I.

### ORIGINAL COMMUNICATIONS.

*Case of remarkable Organic Disease of the Stomach and neighbouring Parts, with the Appearances on Dissection.* By ALEXANDER DEWAR, Esq. Surgeon of H. M. S. Boyne, Flag Ship of Admiral Lord Exmouth.

(Communicated by Dr. Denmark, Physician to the Mediterranean Fleet.)

RICHARD JOLLY, ætatis 32, of a spare habit and sallow complexion, complained, on the 29th of August, 1815, of being affected with nausea, uneasiness at the præcordia, bad taste in the mouth, and loss of appetite; the pulse, skin, and tongue, were natural. He had suffered occasionally from dyspeptic symptoms, for three months previously to this period; but they had given him so little uneasiness, that he had not found it necessary to make his complaints known. About two years before this time, he received an extensive fracture of the right side of the os frontis by a fall: since his recovery from this accident, until the occurrence of the above complaints, he enjoyed good health. An emetic was administered; the bowels were opened by a mild laxative; and tonics, with bitters, were afterwards given. About the middle of September he began to vomit frequently after eating; and, in addition to the other symptoms, he was frequently distressed by acute shooting pains in the forehead, with vertigo. Between this period and the 10th of October, he took, at different times, and variously combined, infusions of quassia, cinchona, and chamomile; tincture of muriate of iron, sulphuric acid; and, as the bowels were habitually costive, laxatives, generally of rhubarb, and submuriate of mercury, or carbonate of magnesia. Blisters were applied to the temples, nape of the neck, and præcordia. The affection of the head was relieved by these means, but the other symptoms had gradually increased in severity. Nothing unusual could be detected by the most careful examination of the epigastrium, nor did moderate pressure increase his uneasiness.

On the 10th of October, an alterative course of submuriate of mercury was commenced, and a small quantity of mercurial ointment was rubbed on the epigastrium daily. The occasional use of tonics and laxatives was continued.

On the 13th of October, he complained that, in swallowing liquids, he felt as though they were stopped, for a time, about the lower part of the œsophagus, and the act of swallowing was followed by a painful hawking of mucus from the pharynx. He did not, at this time, experience such uneasiness in swallowing solids.

On the 20th of October, the symptoms were considerably alleviated: he ate with tolerable appetite; swallowed with greater ease; retained his food; and the bowels were become more regular. This favourable state continued until the beginning of November, when the former symptoms again returned. At this time, also, the right inguinal glands became affected with pain and considerable swelling, and the leg became œdematous. The stools were white, like those passed in jaundice; and continued so until the middle of November, when they became more natural. In the course of a few days, the affection of the right inguinal glands disappeared, and those on the left side became affected in a similar manner. From this time the symptoms gradually advanced; the difficulty of swallowing increased; and the long-continued hawking, which followed deglutition, distressed him so much, as to deter him from making the attempt frequently: what did enter the stomach was generally rejected immediately, mixed with a quantity of ropy mucus. Urgent thirst was a distressing symptom, which he relieved, in some degree, by washing the mouth with cool liquids. During the last three weeks of his life, injections of strong beef tea, or chicken broth, were given every three hours, during the day; 130 drops of tincture of opium being added to the last one given at night. They were always retained; and, although the quantity swallowed at this time was very trifling, and that apparently entirely rejected by vomiting, he passed every day, or every second day, stools of natural appearance and consistence, although in small quantity. Until within two days of his death, the velocity of the pulse was scarcely accelerated; he had no febrile heat; and the tongue continued clean, although latterly the taste was depraved, as he complained of every thing which he took into his mouth being sour. He continued perfectly sensible until within a few hours of his death, which happened on the 15th of December, 1815.

### SECTIO CADAVERIS.

A small quantity of serum was found in the left cavity of the pleura: the quantity of fluid in the pericardium was also greater than usual. The left lung adhered firmly throughout its whole inferior surface to the diaphragm.

On opening the abdomen, lengthened adhesions were found between the omentum and peritonæum, on the right side; and also between the omentum and edge of the liver. The stomach was moderately distended, and its whole upper surface adhered firmly to the liver. Along its great curvature, there were many bodies resembling enlarged lymphatic glands. On grasping the parietes of the stomach, there seemed to be numerous tumors attached to the internal surface, at both its extremities. The pancreas was enlarged and indurated: its left extremity adhered firmly to the stomach and spleen. In attempting to separate this adhesion, an abscess was opened in its substance, which contained about two ounces of thin pus. The great extremity of the stomach, the left extremity of the pancreas, and the parts of the spleen and diaphragm contiguous to them, formed so confused a mass of disease, that no trace of their natural structure could be distinguished.

While trying to unravel the organization of this mass, the cavity of the stomach was accidentally opened; and its contents, consisting of a few ounces of brownish matter, escaped. For about three inches around the cardiac orifice of the stomach, its substance was an inch in thickness, and had a whitish fatty appearance when cut into. The internal surface was covered with loose flocculi, which were easily detached. In one place, where the stomach had given way, in the attempt to separate the parts, it appeared in a state of gangrene. The disease extended about an inch along the canal of the œsophagus; the lower part of which had the same appearance as that in the stomach. But, at the upper part, it formed distinct fungous tumors of various sizes, firm to the feel, with rough surfaces; the largest resembling, in shape and appearance, a pale strawberry. The passage was reduced to a very narrow space; the vessels of the mucous coat, to some distance from the tumors were unusually distinct.

About an inch from the pylorus, towards the small curvature of the stomach, there was a firm fungous tumor, about an inch and a half in length, half an inch in breadth, and three-quarters of an inch in thickness. On its surface, it was divided into different lobuli; but they arose from the same base. The orifice of the pylorus was contracted so as to admit, with difficulty, the large end of a common blow-pipe. The duodenum partook in this contraction for about an inch, and its coats were much thickened. In that part of the stomach unoccupied by the disease in its advanced stage, there were numerous small tumors between the villous and muscular coats; they varied in size, from that of a garden pea to a large pin's head: the largest were found nearest the diseased part of the great extremity. The spleen was smaller than usual, and its substance more firm: its coverings were much thickened; and, in hardness, approached to that of cartilage. A portion of the colon, about an inch in length, on the left side, was contracted to one-third of its diameter. The gall-bladder was much distended, and contained about six ounces of thin serous fluid, slightly tinged with yellow, and a quantity of black gritty particles, of the size of the grains of common gunpowder. The cystic duct was contracted, while the hepatic and common ducts were more capacious than usual.

---

### Observations by the Editors.

Dr. Baillie, in his Morbid Anatomy of the Stomach, describes nothing to which the foregoing disease can be referred.—" I have seen," says he, " several instances of a scirrhous tumor being formed in the stomach, about the size of a walnut, while every other part of it was healthy.' 146.—And again, " tumors consisting of a fatty substance, have been sometimes found in the stomach; but they are to be considered as a very rare appearance of disease." 151.—These instances of morbid organization bear but a very faint similitude to the case related above.

Morgagni, in the 58th article of his 19th letter, relates the case of a drunken old nurse, who for fifteen months had laboured under dry cough, difficulty of breathing, and continual head-ache, with loss of appetite, pervigilium, &c. On dissection, a round tumor, a pound in weight, was found annexed to the posterior surface of the stomach. This tumor was almost of a cartilaginous hardness, and had no communication with the cavity of the stomach, which was, in other respects, sound.—In his 37th letter, he relates the case of a tumor which projected into the cavity of the stomach, of a white, firm, and compact substance. The pyloric orifice and lower part of the œsophagus were here, however, unusually dilated. The tumor did not appear to injure the patient's health.—In his 39th letter, is a case bearing more analogy to Mr. Dewar's. In the antrum pylori were found a number of small and depressed tubercles, of various figures and magnitudes, the largest not being the size of a small bean: they were firm and white in substance. The pyloric orifice was much contracted, and the coats of the stomach and duodenum much thickened. This man had a variety of other organic diseases; and therefore, the symptoms of this peculiar state of the stomach could not be distinguished.—In letter 55, Morgagni makes mention of a man who, after taking some remedies for a gonorrhœa, became affected with gastric irritability; vomiting; anxiety, and pain in the region of the stomach before vomiting; singultus; flow of saliva from the mouth; constipation. No pyrexia, but general wasting of the body till death closed the scene. "The stomach had its pylorus in a state of contraction, and very hard: near the pylorus was a small ulcer; and on the remaining part of the internal surface, were a great number of glands, as it were, scattered at some distance."

In letter 29, Morgagni mentions a stout man, who for some days, was troubled with head-ache, and sense of heat in making water. After a moderate supper, he was seized with violent pains in the region of the stomach, while the head-ache continued as before. The gastric symptoms increasing, a great quantity of green matter was discharged from the intestines, and also by the mouth. He died on the third day. When the stomach was opened, Morgagni discovered a number of small bodies resembling lenticular glands, on the internal surface, with marks of inflammation and incipient gangrene.

Upon the whole, there is probably not a more remarkable case upon record, of organic disease of so many important parts going the length which it here did before the termination of life. Is it not probable that the disease commenced in the tumor near the pylorus — then attacked the structure of the pyloric orifice, et hinc, &c. &c.?

The Editors are much obliged to Mr. Dewar and Dr. Denmark for this interesting case, as they are particularly solicitous to excite the attention of medical men to the study of morbid anatomy, which is, even in this inquiring age, too much neglected.

---

